# Where, when, and how FAIR do data from aquatic mesocosm experiments get published?

**DOI:** 10.1093/biosci/biaf081

**Published:** 2025-07-21

**Authors:** Heidrun Feuchtmayr, Kelly Le Quesne, A Katharina Makower

**Affiliations:** Aquatic Ecosystems Group, UK Centre for Ecology & Hydrology, Lancaster, England, United Kingdom; Aquatic Ecosystems Group, UK Centre for Ecology & Hydrology, Lancaster, England, United Kingdom; Leibniz-Institute of Freshwater Ecology and Inland Fisheries, Stechlin, Germany

**Keywords:** open access, open science, data on request, data availability statement, environmental data

## Abstract

We analyzed 192 publications from two EU projects focused on aquatic mesocosm facilities for a) the number of data publications in a repository on its own and the number of data publications associated with a scientific paper, b) the time lag between mesocosm experiments and data or paper publication, and c) adherence of scientific papers to FAIR principles of data publication. More data sets were published alongside scientific papers (103) than in a data repository alone (17). The time lag between experimental end to paper publication (34.9 months) was not significantly different from the time lag between experimental end to data publication (36.7 months). Regarding FAIR principles, 32.6% of papers achieved the highest scores (7 or 8), emphasizing a high data transparency relative to other disciplines. To improve data publications, we recommend increased support (especially for interoperability) for and recognition of researchers, as well as increased efforts by journals, repositories, and funders.

There has been considerable movement toward open online access to academic publications and data to ensure that information is accessible free of charge (see, e.g., the recommendations by the Royal Society; Boulton et al. 2012). This has been partly because of the democratization and increasing transparency of science but also because most research is funded by taxpayers’ money, and the Internet has fundamentally changed the way we can access information. This applies to open-access scientific journal article publications, which we will refer to as *scientific paper publications* from now on, as well as open data, referred to as *published data sets* from now on.

Funding organizations are increasingly mandating open-access publishing (Piwowar et al. 2018) and making explicit funds available to do so (e.g., the Bill and Melinda Gates Foundation, www.gatesfoundation.org/How-WeWork/General-Information/Open-Access-Policy; the European Commission, https://ec.europa.eu/research/participants/data/ref/h2020/grants_manual/hi/oa_pilot/h2020-hi-oa-pilot-guide_en.pdf; the US National Science Foundation, www.nsf.gov/pubs/2015/nsf15052/nsf15052.pdf; the Wellcome Trust, https://wellcome.ac.uk/pressrelease/wellcome-trust-strengthens-its-open-access-policy; UK Research and Innovation, www.ukri.org/manage-your-award/publishing-your-research-findings/open-access-funding-and-reporting). This has caused a huge change in the publishing landscape, with an increasing number of scientific paper publications and data sets being published open access since the late 1990s, reaching 45% in 2015 for journal articles (Piwowar et al. 2018). In a 2019 study, 156 open government data initiatives were identified across 61 countries, illustrating rapid progress on this front at a global scale (Roche et al. 2020). However, the report on The State of Open Data survey (Hahnel et al. 2023) capturing the motivations, challenges, perceptions, and behaviors of researchers toward open data highlights that more needs to be done when it comes to open access data.

Data may take many forms, including samples, software, field notes, model code, instrument calibrations, genetic barcode, and archival records (see Borgman 2012). In the present article, we focus on samples of large-scale aquatic mesocosm experiments (see below). There are various ways that data can be published open access. Data sets can be published together with a scientific paper publication, either in a data repository (e.g., PANGAEA, Zenodo/Dryad) or in the supplemental material of an open access (i.e., no subscription fee required) scientific paper publication. However, a data set can also be made available in a data repository on its own (i.e., before a journal article is available or with no association to a journal article). There are also data journals available where data sets can be described and published in an article (Beardsley 2014).

Obviously, the more people that are able to access data publications, the wider the information is disseminated and the more it can be reused on a global scale without limitations by any political or administrative borders. The benefits or motivation for researchers for sharing data are numerous (Borgman 2012, Michener 2015, Hahnel et al. 2023, Popkin 2019): safeguarding of data (avoid data loss) either driven by the scientist or as a requirement by the funder or institute; accelerating scientific discovery and innovation; accelerating a career by citation of a journal article or potential co-authorship by catalyzing new collaborations, increased impact and visibility of research, consideration in job review and funding applications; financial reward; saving time and money because data is not recollected multiple times; generating goodwill among researchers; and giving back something of value to taxpayers, such as greater transparency, reuse, reproduced or verified research, and general public benefit, along with increased public trust.

Regarding the benefits for a scientific career, a survey of 140 Canadian researchers in ecology and evolution showed that 47.9% stated benefits from openly sharing data, 43.6% neutral outcomes and only 21.4% stated costs (Soeharjono and Roche 2021). However, in a global survey of 6091 researchers on open data (Hahnel et al. 2023), only around 15% of the researchers stated that they do get sufficient credit for sharing data, whereas 60% did not feel they receive appropriate credit for openly sharing their data.

Credit for sharing data is mostly given as a citation in another article (39%) or co-authorship on a journal article using the published data (23%; Hahnel et al. 2023). This is based on the data set or journal article having received a unique DOI (digital object identifier), which can be used for citation (see Michener 2015), and on the researchers following the ethics code of citing the origin of a data set instead of using data without citation or acknowledgement. However, many researchers have never received any form of credit and recognition (37%; Hahnel et al. 2023), which might hinder motivation when it comes to making data openly available. The outcomes of a survey of 1329 scientists revealed that 91.7% agreed or strongly agreed to the statement “it is important that my data are cited when used by other researchers,” and 59.7% would expect a co-authorship on a publication resulting from the use of their data, whereas 80.6% would like the opportunity to collaborate on the project (Tenopir et al. 2011). Additional obstacles or costs in making data openly available are the fear of misuse or misinterpretation of the data, being scooped (i.e., somebody publishes a journal article using the data before the data provider), the time effort required to document the data (i.e., writing a metadata or readme file), control over intellectual property, a lack of funding to make data available, a lack of data management support, and the possibility of the scientist providing the data not being given a co-authorship on journal articles using their data (Tenopir et al. 2011, Borgman 2012, Soeharjono and Roche 2021).

However, there seems to be a large proportion of researchers who agree with the benefits of data sharing and want to make their data available but lack support to do so. A report (Hahnel et al. 2023) highlighted that more needs to be done in terms of supporting researchers with planning, managing, or sharing their data in order to overcome the obstacle of data sharing. Although Roche and colleagues (2020) acknowledged the lack of background or training of scientists in data management, they provided plenty of information and open access resources with guidance for managing and publishing open data (e.g., on data management, data formatting and protection and metadata documentation), including open access online training resources for data management. The importance with any data publication is to comply with the FAIR (*findable, accessible, interoperable, reusable*) data principles to ensure reusability of the data and emphasizes that the data need to be findable and usable by machines (Wilkinson et al. 2016). There are also other principles to consider such as CARE (*collective benefit, authority to control, responsibility, ethics*) principles for Indigenous data governance (Carroll et al. 2020), but in this study, we focused on FAIR data principles. Scientific paper publications complying with FAIR principles are currently not the norm (e.g., less than 1% of 306 studies in cancer related articles indexed in PubMed in 2019; Hamilton et al. 2022); however, 83.5% of researchers agree or strongly agree with using other researchers’ data sets if their data were easily accessible (Tenopir et al. 2011).

Another obstacle for many researchers is time pressure. Apart from writing a metadata or readme file, the publication of data into a data repository is often reasonably straightforward. Borgman (2012) stated that researchers must choose carefully where to spend their time and resources. If the advantages of data sharing are not a high priority, data sharing might occur only whenever a journal article is published and the journal requires data availability statements. Therefore, data are often only made available together with a journal article, which can take a considerable time after a project has finished. A similar time lag can occur where researchers are worried that by sharing their data without a journal article, they will not be the first ones to publish a journal article on the data (Popkin 2019). It is currently unclear whether this time lag creates a problem for researchers as monetary funds for open access publications or journal publication fees (article processing charges) are often available within a project but have to be spent before the project finishes.

In the present article, as a case study, we analyzed data sets and scientific papers from two consecutive European Union–funded projects uniting researchers from 16 European countries: AQUACOSM and AQUACOSM-plus. Both projects were focused on large-scale aquatic mesocosm experiments, usually intensively sampled for a defined amount of time, and therefore providing a unique opportunity to explore if data are shared (FAIRly). To our knowledge, no publication so far has investigated how open access data sets are published (with or without a scientific paper) within large projects over time. With increased support and knowledge of the benefits of open access, we aimed to assess whether researchers are publishing more data sets in repositories without a link to a scientific paper and increased FAIRness. Understanding which of the four FAIR principles were most followed or which principle would need future improvement can provide guidance for large project consortiums in other research areas. Furthermore, because large-scale experiments are often labor and cost intensive, they are defined with a clear end date and therefore provide the opportunity to compare the time span between experiment and scientific paper publication. Focusing on those time lags from large-scale experiments allowed us to investigate whether open access publication fund, often provided by funders only in the lifetime of the project, can be used effectively. To our knowledge, this has not been done before, likely because of a lack of suitable projects including a large number of well-defined experiments.

The research questions we aimed to address in the present article were these: Are data from mesocosm experiments primarily published in a data repository or as part of a scientific paper and does this change over time with increased support and education? Is the time lag between mesocosm experiment and data publication in a repository shorter than the time lag between experiment and data publication linked to a scientific paper and can open access funds be used effectively? How FAIR are the data from open access scientific paper publications on mesocosm experiments?

## Methods

The AQUACOSM and AQUACOSM-plus projects involved a network of aquatic mesocosm facilities across Europe from 2017–2020 and 2020–2024, including 21 and 30 partners, respectively, and over 60 mesocosm facilities. Both projects were focused on providing funding for transnational access users to take part in aquatic mesocosm experiments in countries other than their home countries, maximizing new international collaborations but also knowledge transfer. The transnational access users included students, as well as researchers, who are interested in mesocosm experiments and applied to take part in mesocosm projects published on the webpage of AQUACOSM or AQUACOSM-plus or suggested their own ideas for mesocosm research. A panel of scientists not directly involved in AQUACOSM and AQUACOSM-plus then ranked the applications, and if they were successful, transnational access users conducted research at mesocosm facilities with travel and subsistence paid for by the AQUACOSM and AQUACOSM-plus projects. The data sets and scientific paper publications reported below were published by all scientists working with mesocosm experiments, transnational access users, and mesocosm facility providers. Mesocosm facility providers were project partners within AQUACOSM and AQUACOSM-plus who provided a mesocosm facility for experimental studies and the transnational access users.

### Data publications from AQUACOSM and AQUACOSM-plus project published open access

**Figure 1.**
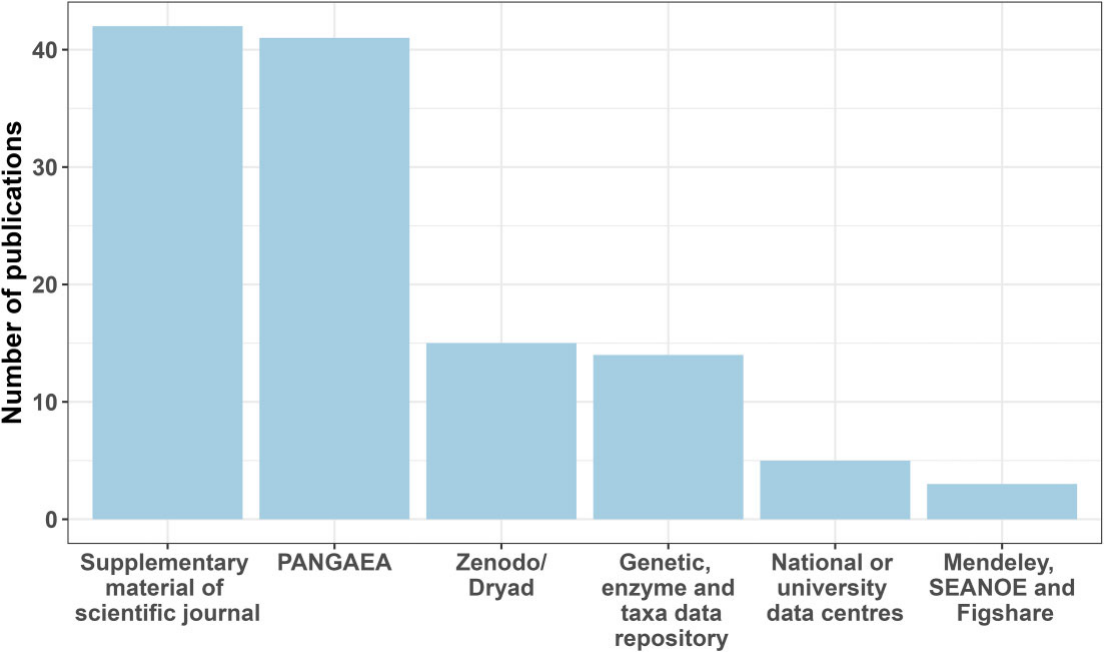
Number of data sets published from the AQUACOSM or AQUACOSM-plus project by location of publication (ordered highest to lowest). The genetic, enzyme, and taxa data category consists of five different repositories: NCBI, the European Nucleotide Archive, EMBL-EBI Metabolights, Ecotaxa Obs, and MassIVE. The national archives or data centers include Danish ODA and LAGOSNE, NERC EDS EIDC, Research Data UNIPD, and the Swedish National Data Service.

**Figure 2.**
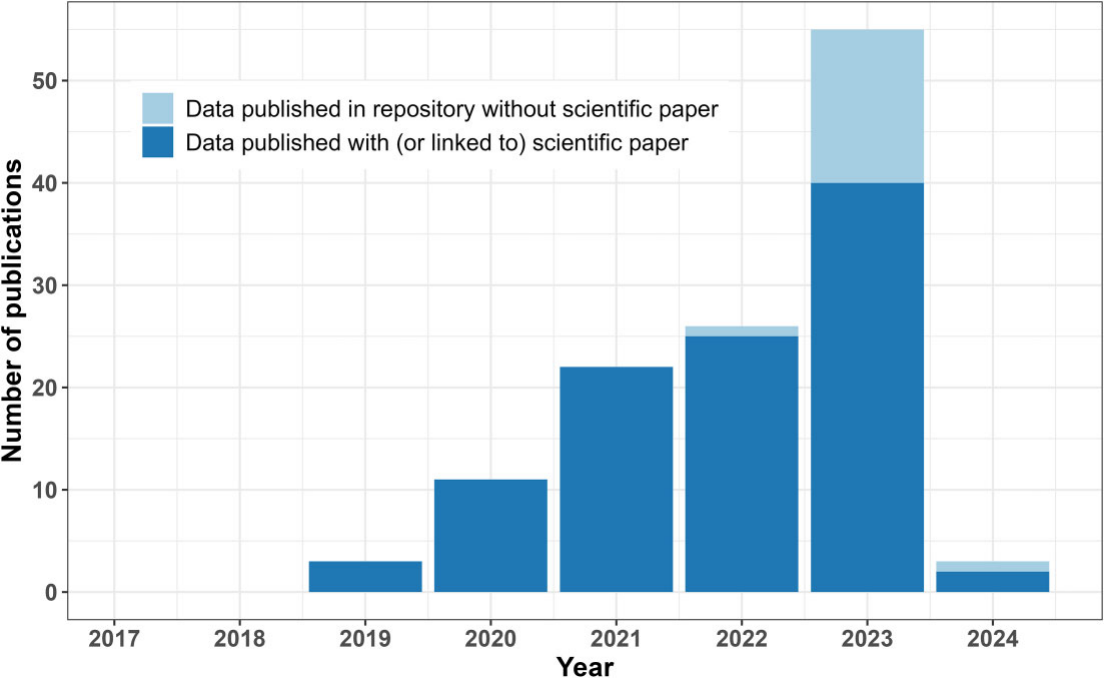
The number of data sets published from the AQUACOSM or AQUACOSM-plus project for all years (*N* = 120) together with a journal article in data repository or with at least some data in the supplementary material (the dark bars), or published in a data repository without link to a journal article (the light bars). The 2024 data include only January to mid-March.

In order to obtain a complete list of data published within AQUACOSM and AQUACOSM-plus, we searched for data sets (or links to data sets) within journal articles using Web of Science and Google Scholar and within data repositories (PANGAEA, Dryad/Zenodo, EmodNet, SEANOE, Mendeley), as well as data holdings for genetic, enzyme, and taxa data (NCBI, European Nucleotide Archive, EMBL-EBI Metabolights, Ecotaxa Obs, MassIVE). The search terms we used were *AQUACOSM* and *AQUACOSM-plus*. A cross-check was performed to identify any data sets linked to journal articles missed in our search and vice versa. The paper publications were manually checked for data availability by looking at the paper, supplementary materials and their data availability statements. Journal articles that did provide some data in the paper or their supplementary material were included in figure 1 and 2. Journal articles that contained data in the supplemental material and in a data repository were considered a single item in figures 1 and 2 and were categorized as data in repository. For a complete list of open access data sets and scientific paper publications, including DOIs, please see the table in the data repository given in the data availability statement.

To understand the number of data sets and data set titles that are planned on being published from AQUACOSM and AQUACOSM-plus in the near future, all of the facility providers were asked via email to populate an online spreadsheet and to contact their transnational access users to incorporate transnational access data sets.

### The time lag between mesocosm experiments and data set or scientific paper publications

The duration of data set publication was determined as the time span (as the number of months) between the last month of the last experiment and the month of publication of the scientific paper or data set. The mesocosm facilities within AQUACOSM and AQUACOSM-plus were all large scale, and the experiments were clearly defined with a start and end date, either provided in the metadata of data sets or the method section of journal articles. For most experiments, the end date of an experiment coincides with the last day of sampling. For repositories, the data set publication date was taken from the date the data were uploaded into the repository, and the end of the experiment was determined by manually checking though all data set metadata and sometimes the data itself. For journal articles, the publication date was obtained from the first page of the journal article or the details given on the journal’s webpage and the “Methods” section of the journal articles. The time lag analysis included journal articles where the data were made available via open access, as well as via direct request (please see the table in the data repository given in the data availability statement). Experiments that ran before AQUACOSM started (i.e., 2017) were removed from the time lag analysis, even though the data or journal article was published during the run time of AQUACOSM or AQUACOSM-plus. Journal articles not reporting on experimental results (e.g., meta-analyses) were excluded from the time lag analysis, along with articles where no experimental dates were given. A Mann–Whitney *U* test (nlme package; R Core Team 2024) was performed to determine whether there was a statistically significant difference between the time lag of experiment to journal article and the time lag of experiment to data set publication (without journal article).

### FAIRness of data publications from open access scientific paper publications

In order to determine whether the data from open access scientific paper publications were published and were published FAIR, we adopted the DATA (for *discoverable, accessible, transparent*, and *actionable*) scoring criteria from Van Tuyl and Whitmire (2016; see their table 1) for the FAIR principles (table 1) and manually scored each paper accordingly. Each article was scanned for information about shared data in the acknowledgements and supplemental material, any data links were followed, and data were downloaded and checked for completeness (compared with figures in the “Results” section). To the best of their ability, the same person scored all of the scientific papers. The use of shared vocabulary was determined as usage of common vocabulary by the present authors; however, uncommon or unclear parameters or abbreviations were checked with EnvThes (controlled vocabulary developed in the context of LTER Europe for ecological research, monitoring, and experiments; https://vocabs.lter-europe.net/envthes/en). All of the scores were summed to determine how many scientific paper publications reached the highest scores of 7 and 8.

**Table 1.** Scoring criteria for the effectiveness of data sharing from Van Tuyl and Whitmire (2016; see their table 1), adopted for journal article scoring of shared data under the FAIR principles.

Effectiveness	Score	Criteria
Findable	0	No link or indication of data source in the paper OR non-actionable mention of data location (e.g., broken link, mention of source without link) OR no raw data sharing
	1	A reference to the location or source of the data but no specific indication of the data used (e.g., link to researchers’ home page or external database) OR data shared are not all of the data used in the paper OR summarised data instead of raw data is presented.
	2	A direct link to a data set with a persistent identifier (e.g., DOI) OR all data provided in supplemental material OR link to open access genetic database
Accessible	0	Data shared through closed or subscription access platform or accessed by request OR no raw data sharing
	1	Data shared through a platform that requires some barrier to access—for example, obtaining permission to use the data or closed-access source (e.g., journal, repository)
	2	Data shared in an open repository or platform or source (e.g., supplemental material of open access scientific publication)
Interoperable	0	No documentation or metadata provided for the data OR no raw data sharing
	1	Some documentation or metadata provided for the data but lacks detail (e.g., how data was collected, analysed or processed, description of units) OR metadata provided in journal article OR terminology cannot be verified
	2	Detailed metadata or read me file; using shared vocabulary (i.e., is the vocabulary accessible for humans and machines)
Reusable	0	Data are not in a format that is usable in an analysis application (e.g., PDF or figure) OR no raw data sharing
	1	Data are in a usable format in an analysis application but are formatted in a way that makes use difficult OR data are shared in a nonopen format (e.g., .xls, .doc, .sas, .mat, .shx) OR data in open format but large proportion of data missing
	2	Data are in an open or nonproprietary format (e.g., .csv, .xlsx, .txt) with usable formatting OR genetic data deposited in open access genetic database

**Table 2.** Overview of the number of data sets analyzed for each analysis and the number of data sets excluded from each analysis (for exclusion reasons, please see the text).

Research question	Total number of papers or data sets analyzed	**Scientific papers**	Data sets in repositories	Number of items excluded	Figure number
Open access	120	103	17	72	1 and 2
Time lag	126	73	53	66	3
FAIR scoring	92	92	–	12 (and 88 data sets)	4 and 5

### Results for data publications from AQUACOSM and AQUACOSM-plus project published open access

In total, 120 data sets were published with open access from AQUACOSM or AQUACOSM-plus from 2017 until mid-March 2024 (see table in the data repository given in the data availability statement), 2 weeks before the end of the AQUACOSM-plus project. Most of the data sets were published in PANGAEA and Dryad/Zenodo repositories (56 data sets); 42 of the data sets were published in tables in the supplemental material of scientific journals; 14 of the data sets for genetic, enzyme, and taxa data were published in five different data holdings (NCBI, European Nucleotide Archive, EMBL-EBI Metabolights, Ecotaxa Obs, MassIVE), which often need specialized data repositories, whereas three data sets were published in three other repositories (Mendeley data, SEANOE, and Figshare; see figure 1). Data were also deposited in national archives or data centers (Danish ODA and LAGOSNE, NERC EDS EIDC, Research Data UNIPD and Swedish National Data Service) because some universities or research institutes have internal (or national) guidelines they are advised to follow (5 data sets). In addition to the 120 open access data sets published, 25 data sets associated with open access journal articles are available on request, so although the journal is open access, we did not consider the data to be accessible and did not use these data sets in the above analysis. Other reasons for exclusion were that the experiment finished prior to AQUACOSM (9 data sets), no data was generated or available (8 studies, e.g. model studies), the DOI link was not working (*n* = 1), or to avoid duplication, i.e. that the paper was linked to other data sets which had already been included in the analysis (*n* = 29).

Our analysis showed that most data sets were published with or linked to a journal article (figure 2: 103 data sets compared with 17 data sets published without a link to a journal article). Only three of the data sets were published before 2020, and most of the data sets were published in 2023, either in a repository without a link to a scientific paper or together with or linked to a scientific paper.

From 27 AQUACOSM-plus facility providers, the spreadsheet regarding future data set publications acknowledged 24 different facility providers. Within the next couple of years, there are 82 data sets planned to be published, either by the facility providers themselves or by transnational access users, which adds up to 35% of the total available data sets from both projects. These data are an estimation, and information on how (with a journal article or without) or where the data sets will be published is currently unavailable.

### Results for the time lag between mesocosm experiments and data set or scientific paper publications

**Figure 3.**
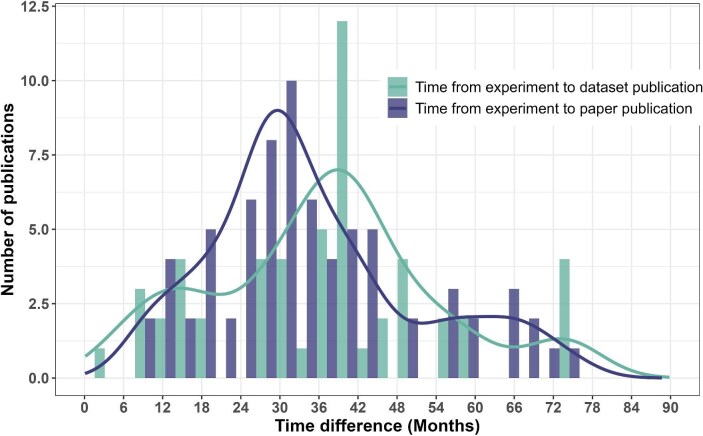
The time difference (in months) of data sets published from the AQUACOSM or AQUACOSM-plus project (in data repository or supplemental material) together with a journal article (the dark bars) or published in a data repository without link to a journal article (the light bars), shown with density lines.

Overall, a total of 126 data sets were used for this analysis. For data sets published in a data repository, 53 datasets could be used for the time lag determination, while 11 were excluded because the experiment either finished prior to AQUACOSM (*n* = 2), or the data were not from a mesocosm experiment (*n* = 4), or there were no dates given (*n* = 5). For data sets associated with scientific papers acknowledging AQUACOSM or AQUACOSM-plus, 73 were used in the time lag comparison. This included 35 scientific papers publishing their data together with the paper, and 17 scientific papers whose data availability statements stated “data available on request”, 20 papers where data was published in a repository, and one paper without any data in supplementary material (figure 3). If a single data set was used for multiple papers, the time lag for each paper was determined individually. Where there was one scientific paper that used multiple data sets, this was counted as a single time lag for a scientific paper, and the (multiple) data sets were not included (*n* = 26). Six scientific papers were excluded that did not contain exact month or year of running the mesocosm or associated laboratory experiment, and an additional 16 scientific papers were omitted that were not an experiment (e.g., reviews or meta-analyses). An additional seven scientific papers were removed as the mesocosm experiments reported on were conducted before the start of AQUACOSM.

The average time between the end of a mesocosm experiment and the publication of a scientific paper was 34.9 months, and the average time between the end of a mesocosm experiment and a data set publication was 36.7 months (figure 3). There was no significant difference between the two distributions (Mann–Whitney *U* test, *p* = .3).

### Results for FAIRness of data publications from open access scientific paper publications

**Figure 4.**
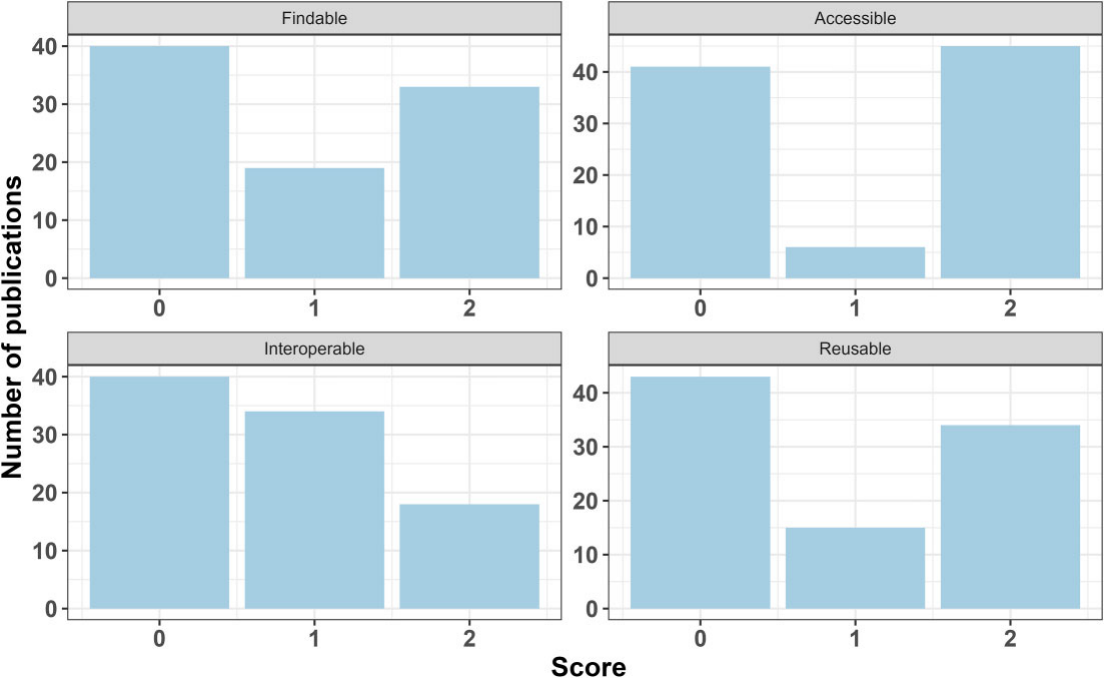
FAIR (findable, accessible, interoperable, reusable) scores from scientific paper publications from the AQUACOSM or AQUACOSM-plus project.

**Figure 5.**
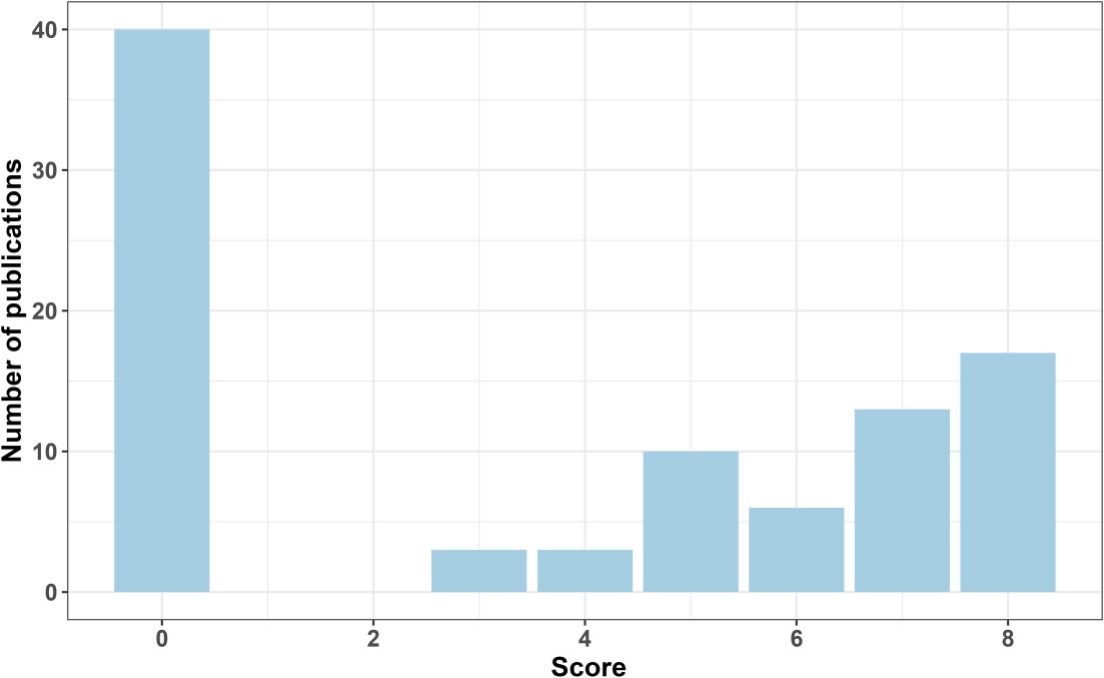
Total FAIR (findable, accessible, interoperable, reusable) scores from scientific paper publications from the AQUACOSM or AQUACOSM-plus project.

The adapted FAIR principles scoring criteria (table 1) from Van Tuyl and Whitmire (2016; see their table 1) was performed on 92 scientific papers and scorings for findability, accessibility, interoperability, and reusability were assigned to every paper (figures 4 and 5). The papers which were not part of AQUACOSM (*n* = 6) and papers which did not include any data (*n* = 6: three modelling studies, one framework and one review that did not generate any data, and one paper which re-visited a publication which did not generate data) were excluded along with dataset publications (*n* = 88). Overall, most of the scientific papers did not adhere to the FAIR principles and scored a 0 for each criterion (figures 4 and 5). However, when looking at each criterion (figure 4), a large number of papers shared their data in ways that were highly findable, accessible, and reusable. There were a couple of intermediate scores (a score of 1), mostly because of not sharing all the data used in a paper, especially important data to reproduce the results. For interoperability, the distribution looked slightly different, with fewer papers scoring 2. A high number of scientific papers did not document the data or did not use well defined vocabulary, and the paper and its method section is needed to be able to understand the data set.

A total score of 0—that is, papers with inaccessible or unavailable data—was found for 40 paper publications (figure 5), but individually, 35.9%, 48.9%, 19.6%, and 36.9% of the paper publications achieved a score of 2 (figure 4) for findability, accessibility, interoperability, and reusability, respectively. A total score of 8 was achieved by 18.5%, and a total score of 7 was achieved by 14.1% of the papers (figure 5).

## Discussion

Given the requirement to adhere to the open data policies of the European Commission, project partners (especially researchers in the Data Work Package of the AQUACOSM and AQUACOSM-plus project) have actively educated, encouraged, and supported all mesocosm facility providers, early-career researchers, and transnational access users to publish their data openly, comply with FAIR data principles and ensure metadata homogeneity. This was achieved via presentations on open access and FAIR principles at general assemblies, project meetings, spring and summer schools, online meetings, videos on the project webpage, and various project deliverables openly available to project partners. Regarding the FAIRness of data, the AQUACOSM and AQUACOSM-plus strategy documentation states that data should be easy to access, distribute, and reuse and emphasizes best practices in reducing (meta)data heterogeneity in various deliverables. AQUACOSM and AQUACOSM-plus also encouraged all mesocosm researchers to make primary data openly accessible within 6 months after completion of the publishable data set, with reasons given if this is not possible. The publishable data set is defined as a data set that has been subject to processing routines aimed at quality assurance and quality control, with open tools for data processing. The reasons for not making the publishable data set openly accessible may include such things as the completion of a PhD thesis, in which case a moratorium of up to 3 years is granted.

### Data publications from AQUACOSM and AQUACOSM-plus project published open access

Addressing our first research question, most data sets from mesocosm experiments were published with or linked to a journal article (figure 2). Interestingly, only three data sets were found before 2020, suggesting a rather lengthy time lag between mesocosm measurements and data publication. By the end of August 2020, 171 transnational-access projects had been completed within the AQUACOSM project (data obtained from A. Katharina Makower, AQUACOSM and AQUACOSM-plus project manager, personal communication, 27 March 2024), of which 115 were conducted before 2020 (2018–2019). It is likely that the number of transnational access projects does not translate into the same number of data sets because some projects might result in more than one data set, whereas others do not produce a data set at all, instead focusing on transnational access training and knowledge transfer. For the facility providers or AQUACOSM-plus organizers, it can be difficult to ensure the transnational access users make their data available from another country and university or research institute or that they produce data within an unknown time period: For example, transnational access users could take samples back to their home institute and could generate data months or years after the mesocosm experiment had finished. It is striking that, in the years 2017–2022, no data had been published in repositories and indicates a view that data publication is not a priority, possibly because of a lack of support to publish data or other time constraints (see Tenopir et al. 2011, Borgman 2012, Michener 2015, Popkin 2019, and Soeharjono and Roche 2021). The majority of the data sets were published in 2023, which could indicate a time lag of a couple of years but could also be a result of the increased acceptance and adoption of open science practices (see the effort within AQUACOSM and AQUACOSM-plus above), especially as European open science strategies focused heavily on open science, promoting FAIR principles and implementing national open science policies in 2023 (see https://scienceeurope.org/news/launch-of-new-national-open-science-strategies). Both factors likely played a role: The high number of data sets published in a data repository without a link to a journal article in 2023 compared with previous years indicates an increased adoption of open science practice; the increase in the number of data sets published together with a journal article in 2023 indicates a time lag.

Looking forward, the incentives of open science are numerous, with more credit being given to sharing data openly by, for example, universities and funding agencies, driven by recommendations by, for example, the Royal Society, which stated that assessments of universities research should reward the development of open data on the same scale as journal articles and other publications (see Boulton et al. 2012). As a result, the current movement of a more integrative measure of scientific research impact (as opposed to the commonly used *h*-index; e.g., Koltun and Hafner 2021) provides a great opportunity to give more credit to sharing open data.

### The time lag between mesocosm experiments and data set or scientific paper publications

Addressing our second research question, the average time between the end of a mesocosm experiment and the publication of the related scientific paper was 34.9 months, and the average time between the end of a mesocosm experiment and its data set's publication was 36.7 months. This difference (1.8 months) was not significant (figure 3). Most researchers are well aware that paper publications can be time consuming, including submission (and possible rejection), reviewing, dealing with reviewers’ comments, proofreading, and publication itself. Powell (2016) reported a median time from submission to acceptance of around 100 days and around 25 days from acceptance to publication of all papers in PubMed from approximately 2010 to 2015. Assuming that a scientific paper is not rejected, this leaves around 29 months from the experiment’s end to a final manuscript ready for submission. This number seems high but could indicate the high workload of researchers running large-scale experiments; a prolonged time for international co-authored papers, including transnational access users in various countries; or PhD students focusing on gathering data in their first and second years and concentrating on writing scientific papers in the latter years of their PhD.

Either way, the outcome is rather surprising, because a data set would generally be expected to be published much faster than a scientific paper, given the tasks involved, such as a quality check. Indeed, figure 3 shows that some data sets are published in a data repository within about 18 months. However, on average, the data sets were not published significantly earlier than the scientific papers, potentially because of the time constraints of the researchers only being able to write scientific papers based on part of the data and publishing the rest of the data in a repository.

The time lag of around 3 years from gathering data of large-scale mesocosm experiments to the publication of data sets or journal articles may seem long. However, given the high complexity of large-scale experiments (relative to laboratory experiments), perhaps the 3-year time lag is reasonable. For example, the large number of measured variables in such studies make them complex, as does the very nature of infrastructure EU projects attracting collaborations with people in different countries, including PhD students working on their thesis for usually 3–5 years, depending on the country. The AQUACOSM and AQUACOSM-plus projects each lasted for 4 years. It can easily take 1 year from the start of a project to the end of a mesocosm experiment, with the planning and setting up of the experiment, advertising for transnational access users, and project managing finances. With an additional period of nearly 3 years from the end of an experiment to publishing a journal article, however, the current timeframe to use EU project funding for open access is nearly impossible. This is further emphasized by the outcome of our survey of transnational access users and mesocosm facility providers, which showed that around 35% of the total data sets are yet to be published after the end of the project.

Open access publication can be expensive, ranging from approximately €1.000 to €10.000 per journal article. The Open Research Europe publishing platform provides open access, free of charge research publications from Horizon 2020, Horizon Europe, or Euratom funding. However, the platform contains journal articles from all subject areas, and some researchers might argue that a journal article published in a subject specific journal has a better chance of attracting readers and accumulating citations and impact. Some universities and research institutes also use journal impact factors to measure the scientific success, productivity, and impact of a researcher, making this path to publication even less desirable.

### FAIRness of data publications from open-access scientific paper publications

While we were researching journal articles from AQUACOSM and AQUACOSM-plus (especially data availability statements, links to data repositories and data in supplemental material), it became apparent that the FAIR principles are not always followed when it comes to open access data. Scoring all the scientific papers from both projects on FAIR (findability, accessibility, interoperability, and reusability) principles (addressing our third research question) showed that there are some researchers who take a lot of care and time to ensure the data is FAIR, scoring 2 for most principles (figures 4 and 5), whereas others do not (scoring 0 for most principles; see figures 4 and 5). Apart from the data-on-request statement found in various data accessibility statements required by scientific journals, we also found links to a data repository instead of a link to a specific data set, as well as statements such as “I've shared the link to my data in the manuscript file,” but a link could not be found in the journal article or in the supplemental material. This confirms the findings of Colavizza and colleagues (2020), who analyzed the data availability statements of 531,889 journal articles published by PLOS (the Public Library of Science) and BMC (BioMed Central). Although data availability statements were found in around 90% of all of the journal articles, only 20.8% of the PLOS publications and 12.2% of the BMC publications (from 2017 and 2018) provided a link to data in a repository (Colavizza et al. 2020), emphasizing the fact that, despite the availability statement, data sets are often not available, findable, or accessible. Similarly, Hamilton and colleagues (2022) searched 306 cancer-related articles indexed in PubMed in 2019 and found that one in five studies declared that their data were publicly available; however, when the data availability was investigated, this was only true for 16% of the studies, and less than 1% of the data complied with key FAIR principles. Although our study had a high number of papers with inaccessible or unavailable data (*n* = 40; see figure 5), we found that 48.9% of the papers from AQUACOSM or AQUACOSM-plus scored an overall score of 2 for accessibility (figure 4), and 18.5% of the papers scored an overall score of 8 (figure 5), publishing the full data set used in the study and complying with FAIR principles, according to our definition outlined in table 1. Even though this number might seem small, if we take into account the myriad of obstacles to data sharing mentioned in the introduction and also compare it with other studies (see Van Tuyl and Whitmire 2016 and Hamilton et al. 2022), this is a very positive outcome indeed.

Quite a few AQUACOSM or AQUACOSM-plus scientific papers published only part of the data used in the supplemental materials, or summary data were presented instead of raw data, or the metadata was missing vital information to allow reuse of the data for other research questions. This makes it—likely unintentionally—very difficult to reuse the data, for example, for meta-analysis, and adds to the complexity of combining various different data and data sets from multiple sources, different countries, and different time points (e.g., Roche et al. 2020). Interoperability showed a pattern different from those of the other principles (figure 4), suggesting that researchers who make their data available did not provide adequate metadata or readme files or did not use shared vocabulary. We believe this to be unintentional, and there seems to be a need for more guidance or help for the researchers to be able to improve the interoperability of their data. Unfortunately, most researchers lack support when it comes to FAIR data publishing, and access to support from specialist data managers is often not available (Hahnel et al. 2023). In a survey of 1329 scientists, most of the respondents stated dissatisfaction with long-term data preservation and a lack of data management support to researchers (Tenopir et al. 2011).

Comparing the results from this study with a study on 104 NSF (National Science Foundation) –funded projects at Oregon State University (Van Tuyl and Whitmire 2016), the AQUACOSM or AQUACOSM-plus community seems to be further ahead regarding data sharing, scoring much higher in all categories. As was mentioned above, this might be a direct result of project partners providing training and, therefore, of encouraging, educating, and supporting all mesocosm researchers to publish their data openly, comply with FAIR data principles, and ensure metadata homogeneity. Van Tuyl and Whitmire (2016) stated that papers with high overall scores (using the DATA criteria) did not achieve a total score of 8 because of low accessibility (data being shared closed access) or low transparency (poor documentation either vague or relying on methods section of the paper). Although accessibility was not a major issue for AQUACOSM or AQUACOSM-plus articles, lower transparency correlates with our findings. When comparing the two highest scores of 7 and 8, only 12% of Van Tuyl and Whitmire's (2016) papers achieved this, whereas it was more than doubled in our study (32.6% of papers scored 7 or 8).

## Conclusions 

Using AQUACOSM and AQUACOSM-plus as a case study has shown that increased education on open access and FAIRness of data publication is important and has likely led to an increase of open access publications and publications of data sets in repositories along with FAIR data publications. It also emphasized that researchers need support from data managers, especially in improving interoperability—that is, writing metadata files. We believe this is not only true for mesocosm research but might be applicable to other large projects and disciplines.

### Researchers

In general, there was an increase of open data over time, which might reflect an increased perception of the benefits of open science; however, we believe scientists need more training and support. AQUACOSM-plus has tackled this issue in various ways including talks at general assemblies and within various work package meetings, open data workshops, and multiple deliverables—for example, data management plans and a workflow for allocating DOIs for data sets. Furthermore, transnational access users were obliged to write up metadata of their projects before being able to claim their expenses. The available support to AQUACOSM or AQUACOSM-plus researchers might have led to the increase of data sets published in a repository in 2023 (see figure 2) and the overall higher amount of shared data than in other studies. However, the outcomes indicate more support was needed on best practices and FAIR principles to ensure interoperability. Additional incentives for more open science would be to increase recognition, such as more credit to researchers and more integrative measures of scientific impact (Boulton et al. 2012 and Koltun and Hafner 2021), which might also lead to higher numbers of open data in the future.

### Data repositories and scientific journals

Data repositories should also do more and focus effort on guidelines and checks on metadata information before data sets are accepted and published to ensure FAIR principles are met. Alongside data repositories, funding agencies’ lack of monitoring of researcher's data compliance also needs to be addressed (Anger et al. 2022). In addition to these efforts, we believe that a systematic change of the scientific publishing system would be most effective to ensure open and FAIR data handling. Journals should be more vigilant when it comes to data availability and data availability statements and check compliance with data sharing policies (see Hamilton et al. 2022). Reviewers are already providing a free consultation service on the science and relevance of journal publications; therefore, they probably do not consider data availability checks as part of their remit. It would be beneficial to the scientific community if journals would put more emphasis on data sharing or data availability by providing more advice and support (e.g., see White et al. 2013, Costello and Wieczorek 2014), resulting in scientists following guidelines and having to spend more time on FAIR open data and metadata. In addition, implementing an internal review on data and data availability statements before accepting the journal article for publication could improve the situation even though journal staff are not scientists and might not be able to spot all data shortcomings. Van Tuyl and Whitmore (2016) also pointed out that “data shared inadequately as part of a journal article reflects poorly on both the researcher and the journal,” asking for journals to take some responsibility for ensuring the quality of the shared data. Noor and colleagues (2006) suggested more drastic enforcement measures by journals whose authors do not publish DNA sequences: either flag the author to decline future submissions until the DNA sequence has been released or remove the online publication from the journal's web site after 1 month if the DNA sequence has not been released until compliance is reached.

Some countries or international governing bodies implement national or international data portals (e.g., the United Nations, http://data.un.org the European Union, www.europeandataportal.eu the World Bank, https://data.worldbank.org the United States, www.data.gov the United Kingdom, https://data.gov/.uk), ensure persistence (see, e.g., CoreTrustSeal) and actively commit to open, effective, and transparent data (Roche et al. 2020). However, because of a plethora of different data repositories and portals, the data are not easily findable. Furthermore, some of the portals are not user friendly, and data sharing can occur in a fragmented fashion within, for example, the Canadian government (Roche et al. 2020). We found that, although Dryad and Zenodo announced the launch of their first formal integration in February 2021 (https://blog.datadryad.org/2021/02/08/doing-it-right-a-better-approach-for-software-amp-data), it is rather confusing, because different numbers of data sets can be found in the two repositories. Within this report, we found eight data sets on Zenodo when searching for AQUACOSM or AQUACOSM-plus, but only three of those were also found on Dryad with the same search terms. In short, there is a need to enhance user-friendly findability to overcome the current lack of federated search—that is, to search for and find data within but also across repositories.

Although some scientists might need help when it comes to data management or publication of data (see above and Hahnel et al. 2023), most scientists are more than willing to share their data. However, as was mentioned earlier, the fear or cost of being scooped or the data being misused needs to be addressed, along with the urge to know how the data are being used (see Tenopir et al. 2011). From the present authors’ experience, as well as findings from Tenopir and colleagues (2011), scientists would like to know what their data are used for instead of uploading it into a black box. They are very keen to prevent misinterpretation but also to be involved in future data analysis using their data. We believe the willingness to share data, make more data available, follow FAIR principles, and spend more time and care on metadata and open-access data publication would increase if data repositories would become more “data provider friendly” with advanced functionalities. Examples include sending notifications when a data set has been downloaded and by whom or ensuring that whenever a data set is used the data user needs to contact the data provider. However, these examples might not be compliant with GDPR (General Data Protection Regulation) and might get complicated with provenance chains. Furthermore, scientists could often use the knowledge on how their data sets are used to improve their project impact reporting.

### Funders

EU projects often include funds for partners to cover open access fees. These funds, if unused, cannot be called on after the end of a project, leaving the scientist in a conundrum if they aim for a subject specific journal. It would be beneficial if the funds could be available beyond the end of the project because of the long duration between setting up large-scale experiments, conducting experiments, data analyses and interpretation, and publication acceptance, as was shown in this study. Although it was not possible under the present funding rules, we suggest this should be considered in the future.

## Data Availability

The data from this manuscript are openly available from the UK Data Service ReShare repository, published together with a read me file explaining the data table. Please follow the link https://reshare.ukdataservice.ac.uk/857943/.

## Author Biography

Heidrun Feuchtmayr (feuchtmayr@ceh.ac.uk) is a senior freshwater ecologist as well as a database manager and Kelly Le Quesne (kelleq@ceh.ac.uk) is a freshwater ecology research assistant with the Aquatic Ecosystems Group, UK Centre for Ecology & Hydrology, in Lancaster, England, in the United Kingdom. A. Katharina Makower (katharina.makower@igb-berlin.de) is a postdoc and project coordinator with the Leibniz-Institute of Freshwater Ecology and Inland Fisheries, in Stechlin, Germany.
